# Machine learning for high-risk hospitalization prediction in outpatient individuals with diabetes at a tertiary hospital

**DOI:** 10.20945/2359-4292-2024-0317

**Published:** 2025-04-14

**Authors:** Carolina Deina, Flavio S. Fogliatto, Mateus Augusto dos Reis, Beatriz D. Schaan

**Affiliations:** 1 Universidade Federal do Rio Grande do Sul, Porto Alegre, RS, Brasil; 2 Universidade Feevale, Novo Hamburgo, RS, Brasil; 3 Instituto de Avaliações de Tecnologia em Saúde - Conselho Nacional de Desenvolvimento Científico e Tecnológico, Brasil; 4 Serviço de Endocrinologia, Hospital de Clínicas de Porto Alegre, Porto Alegre, RS, Brasil

**Keywords:** Diabetes mellitus, Hospitalization, Machine learning

## Abstract

**Objective:**

To characterize, via a predictive model using real-world data, patients with diabetes with a heightened probability of hospitalization.

**Methods:**

At the Endocrinology Unit of a tertiary public hospital in Rio Grande do Sul, Brazil, a retrospective cohort study analyzed initial consultations from January 1, 2015, to December 31, 2017, focusing on 617 patients with diabetes. Within this group, 82.98% (512 patients) did not require hospitalization, while 17.02% (105 patients) were hospitalized at least once. Multiple machine learning algorithms were tested, and the combination of XGBoost and Instance Hardness Threshold models displayed the best predictive performance. The SHapley Additive exPlanations method was used for result interpretation.

**Results:**

The most optimal performance was observed by combining the XGBoost and Instance Hardness Threshold models, resulting in the highest sensitivity (0.93) in accurately classifying hospitalization events, with an acceptable area under the curve of 0.72. Key predictive features included the number of outpatient visits, amplitude of estimated glomerular filtration rate, and age (individuals below 24 years old and between 65 to 70 years old had higher hospitalization likelihood).

**Conclusion:**

The proposed model demonstrated high predictive capability and may help to identify patients with diabetes who should be more closely monitored to reduce their risk of hospitalization

## INTRODUCTION

Diabetes mellitus is a disease characterized by chronic hyperglycemia due to the impaired release and action of insulin, as well as a failure to regulate hepatic glucose production ^(1)^. The two most prevalent types are type 1 and type 2, which account for about 10% and nearly 90% of all cases, respectively ^(2)^. The prevalence of diabetes mellitus has been increasing steadily in recent years and, by 2021, about 537 million people were estimated to have this condition ^(3)^. In Brazil, 12% of the population is diagnosed with diabetes ^(4)^, the sixth country with the highest number of adults with diabetes in the world ^(3)^.

Patients with diabetes are at greater risk of developing chronic complications and related diseases and require more access to health services than those without diabetes ^(5^,^6)^, representing one of the leading causes for hospital admissions and outpatient visits. It was estimated that the economic burden in Brazil reached US$ 2.15 billion in 2016, of which 70.6% are indirect costs related to premature deaths, absenteeism, and early retirement. If the growth rate of diabetes prevalence continues in Brazil, the direct and indirect costs of diabetes will be more than double by 2030 (an increase of 133.4% or 6.2% per year) ^(7)^.

Artificial intelligence (AI) is a growing field and its applications to diabetes could reshape the management of this chronic condition. Artificial intelligence algorithms have been used to develop predictive models for the risk of developing diabetes and its complications and to optimize the use of healthcare resources ^(8^,^9^,^10^,^11^,^12)^.

Electronic medical records (EMRs) allow the consistent and homogeneous gathering of data, enabling the repository use to train and develop algorithms ^(10^,^13)^. Such records have been used in medical studies with several objectives, including the prediction of hospitalization using the patient’s first record in the emergency room ^(14)^. The possibility of predicting which patients with diabetes are at greater risk of hospitalization and mortality via their characteristics would lead to early interventions that reduce risk, optimize treatments, and better prepare hospital resources to provide adequate care. Few studies evaluate the prediction of hospitalization of patients with diabetes ^(15^,^16^,^17^,^18)^.

This study aimed to characterize, via a predictive model using real-world data, patients with diabetes with a heightened probability of hospitalization.

## MATERIALS AND METHODS

### Study database

This was a retrospective cohort study using a database composed of EMRs of patients from the outpatient Endocrinology Unit of a tertiary public hospital from Southern Brazil. The complete dataset consists of EMRs of patients who had their first medical appointment in the period between January 1, 2015, and December 31, 2017, totaling 2,973 patients. Data within the 2-year period after the first medical appointment were used. Only patients diagnosed with diabetes were selected. They were identified via information from the International Classification of Diseases (ICD) of the first medical appointment and/or result of the first plasma glucose (≥ 126 mg/ dL) and/or the first glycated hemoglobin (HbA1c) (≥ 6.5%) measurements. The final dataset contained 617 patients, 512 (82.9%) were not hospitalized within the 2-year period and 105 (17.0%) were hospitalized at least once during that period. The EMR contained relevant information such as age, skin color, gender, number of outpatient visits, and laboratory tests (creatinine, plasma glucose, HbA1c, and urinary albumin concentration (UAC). The presence of diabetic kidney disease (DKD) was assessed with UAC and estimated glomerular filtration rate (eGFR) calculation using the CKD-EPI equation ^(19)^. Diabetic kidney disease was defined as an eGFR < 60 mL/min/1.73 m^2^ and/or UAC from a single urinary sample ≥ 14 mg/L ^(20^,^21^,^22)^. All textual records were written in Brazilian Portuguese.

This study was approved by the hospital’s Ethical Committee under number 43431521.0.0000.5327. The data were obtained via anonymized query and the consent form was waived.

### Intelligent system protocol

**Figure 1:**
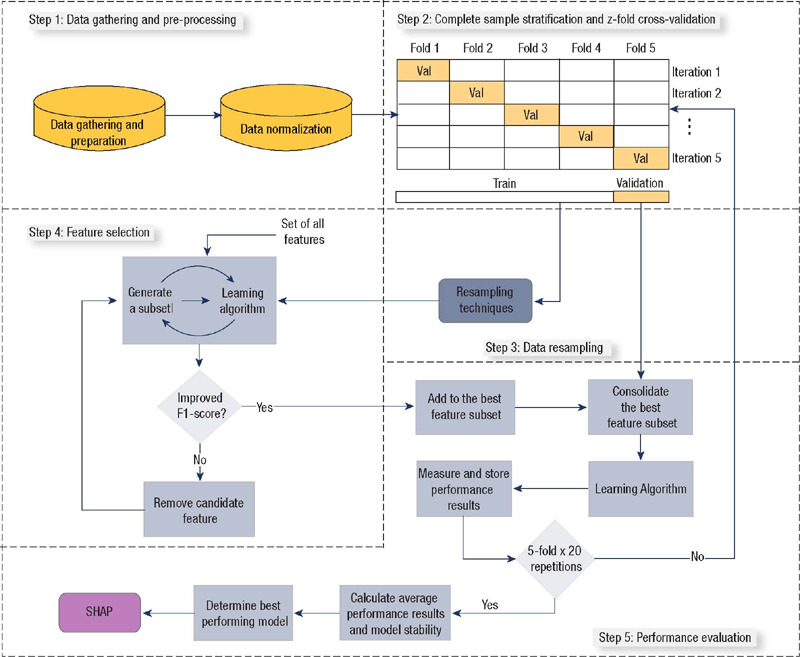
Outline of the proposed method.

We adopted a five-step method (Figure 1) to predict the occurrence of hospitalization in patients with diabetes from their EMRs. In the first step, we gathered, pre-processed the data, and discarded repeated observations. Missing data were imputed using the *k*-Nearest Neighbor method ^(23^,^24)^. After pre-processing, the dataset was rescaled using the max-min scaling in Eqn. 1, the values of all continuous features were in the range [0, 1]. In the equation, *X* represents the feature’s values, and *X_max_* and *X_min_* are the largest and smallest feature values in the dataset.

SHAP: SHapley Additive exPlanations.

Eqn. 1 $$Scaling=\left(X-X_{\min}\right)/\left(X_{\max}-X_{\min}\right)$$

In step two, we divided the complete dataset to obtain train and validation portions using the κ-fold cross-validation technique ^(25)^. The complete dataset is partitioned into five subsets (κ = 5), and the model is trained on four of them while being validated on the remaining subset. This process was iterated five times, ensuring each fold acted as validation set at least once. At each fold, we used a stratified randomized sampling approach to ensure proportional representation of each class (hospitalized and not hospitalized) and reflect the class proportions of the complete sample ^(26)^. To obtain a better generalization of the model, we repeated the five-fold cross-validation process 20 times. In each repetition, we randomly shuffled the dataset before dividing it into five folds for cross-validation. This ensured the model was trained and validated on multiple different combinations of data sets, allowing it to capture more general and robust patterns in the data. At the end of these 20 repetitions, we obtained 100 validation results (five folds × 20 repetitions).

Our goal was to correctly identify patients at risk of hospitalization, i.e., the minority class. Therefore, in step three of the method we applied resampling techniques to the training portion. These techniques are recommended when dealing with highly unbalanced class problems, in which results can be influenced by the majority class. We tested six resampling techniques: Instance Hardness Threshold (IHT), Random Under Sampler (RUS), Synthetic Minority Oversampling Technique (SMOTE), adaptive synthetic sampling (ADASYN), synthetic minority oversampling technique and edited nearest neighbor (SMOTEENN) and synthetic minority oversampling technique with Tomek links technique (SMOTETomek) (Table S1). Resampling was not applied to the validation set, as we wanted to evaluate classification results in a reallife situation.

In step four, we performed feature selection using a wrapper method ^(27)^. In step five, we used the best subset of features in step four in the validation portion of the dataset for each machine learning algorithm tested; they are logistic regression (LR), K-nearest neighbors (KNN), support vector machine (SVM), Extreme Gradient Boosting (XGBoost), and Bagging Classifier using Decision Trees (Table S2). We averaged the one hundred validation results for the following performance metrics: accuracy, positive and negative predictive values (PPV and NPV, respectively), sensitivity, specificity, F1-Score, and are under the Receiver Operating Characteristic (ROC) curve.

We finally selected the model with the best predictive performance and used the SHapley Additive exPlanations (SHAP) method to analyze the results. This involves calculating SHAP values, which are obtained using a game-theoretic approach ^(28)^. The calculation of SHAP values involves evaluating the contribution of each feature to the model’s prediction by comparing the prediction with and without the feature, while considering all possible feature combinations. This process is applied to every feature and observation in the dataset, producing a matrix of SHAP values that reveals the relative importance of each feature to each observation. That enables a deeper understanding of the importance of each feature in the prediction and allows identifying features with the greatest impact on the model’s output ^(29^,^30)^.

### Statistical analysis

A convenience sample was used. The algorithm performance was measured by the area under the ROC curve generated by plotting sensitivity versus one *minus* specificity. Based on the two operating points, 2×2 tables were developed to characterize the sensitivity and specificity of the algorithm. All statistical analyses, methods, techniques, and machine learning algorithms were implemented via Python (version 3.9.12).

## RESULTS

**Figure 2:**
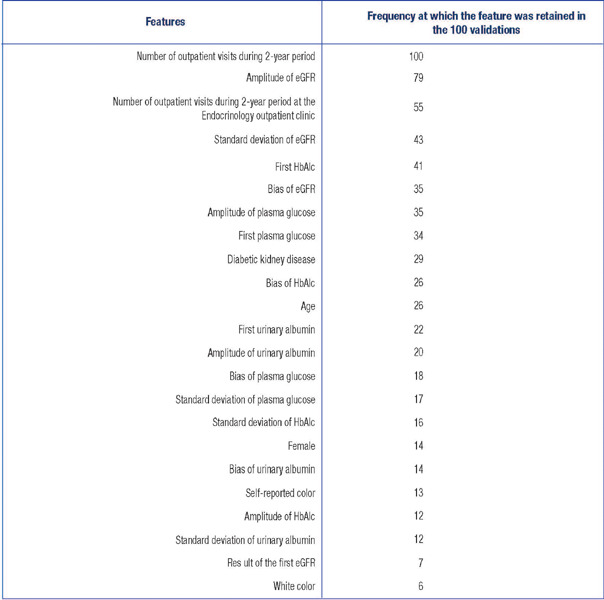
Features used as inputs and frequency with which they were retained in the one hundred validations of the best predictive model.

Figure 2 shows the features used as predictors and the frequency in which they were selected by the best performing classification algorithm. Table 1 reports the validation results, with the best performers for each metric highlighted in bold.

Standard deviation measures the dispersion of data, amplitude represents the range of values, and bias indicates a consistent error or distortion in data. eGFR: estimated glomerular filtration rate; HbA1c: glycated hemoglobin.

**Table 1:** Average predictive performance and standard deviations obtained from one hundred replicates of the dataset validation portion for different combinations of resampling technique and classification algorithm

Resampling technique	Classification algorithm	Performance metrics						
		Area under the ROC curve	Sensitivity	Specificity	Negative predictive value	Positive predictive value	F1_score	Accuracy
Instance Hardness Threshold	LR	0.7280 ± 0.0519	0.6428 ± 0.1044	0.8133 ± 0.0430	0.9179 ± 0.0218	0.4181 ± 0.0604	0.5036 ± 0.0660	0.7842 ± 0.0362
	KNN	0.7141 ± 0.0398	0.8890 ± 0.0816	0.5393 ± 0.0686	0.9611 ± 0.0262	0.2859 ± 0.0289	0.4313 ± 0.0355	0.5988 ± 0.0521
	SVM	0.7099 ± 0.0429	0.8609 ± 0.0705	0.5588 ± 0.0617	0.9518 ± 0.0232	0.2881 ± 0.0311	0.4308 ± 0.0393	0.6102 ± 0.0507
	XGBoost	0.7202 ± 0.0397	**0.9333** ± **0.0538**	0.5071 ± 0.0617	**0.9740** ± **0.0205**	0.2818 ± 0.0284	0.4323 ± 0.0358	0.5797 ± 0.0514
	Bagging	0.7123 ± 0.0373	0.9266 ± 0.0688	0.4979 ± 0.0609	0.9720 ± 0.0244	0.2760 ± 0.0238	0.4247(0.0313	0.5709 ± 0.0477
Random Under Sampler	LR	0.7184 ± 0.0481	0.6028 ± 0.0967	0.8340 ± 0.0383	0.9114 ± 0.0191	0.4317 ± 0.0638	0.4997 ± 0.0652	0.7947 ± 0.0326
	KNN	0.6901 ± 0.0528	0.6680 ± 0.1002	0.7122 ± 0.0521	0.9132 ± 0.0241	0.3257 ± 0.0488	0.4359 ± 0.0581	0.7047 ± 0.0440
	SVM	0.7343 ± 0.0506	0.6623 ± 0.1088	0.8062 ± 0.0524	0.9217 ± 0.0219	0.4190 ± 0.0625	0.5085 ± 0.0636	0.7817 ± 0.0403
	XGBoost	0.7180 ± 0.0589	0.7161 ± 0.1096	0.7198 ± 0.0558	0.9257 ± 0.0270	0.3479 ± 0.0568	0.4660 ± 0.0664	0.7191 ± 0.0481
	Bagging	0.7124 ± 0.0478	0.7128 ± 0.0965	0.7120 ± 0.0495	0.9242 ± 0.0226	0.3394 ± 0.0442	0.4579 ± 0.0526	0.7122 ± 0.0398
Synthetic Minority Oversampling Technique	LR	0.7408 ± 0.0479	0.6442 ± 0.0965	0.8374 ± 0.0357	0.9203 ± 0.0196	0.4524 ± 0.0611	0.5286 ± 0.0636	0.8046 ± 0.0308
	KNN	0.6887 ± 0.0569	0.6147 ± 0.1110	0.7626 ± 0.0516	0.9066 ± 0.0243	0.3513 ± 0.0629	0.4441 ± 0.0696	0.7375 ± 0.0436
	SVM	0.7505 ± 0.0485	0.6609 ± 0.1024	0.8402 ± 0.0375	0.9241 ± 0.0203	0.4636 ± 0.0614	0.5411 ± 0.0630	0.8097 ± 0.0303
	XGBoost	0.6820 ± 0.0511	0.4919 ± 0.0982	0.8721 ± 0.0333	0.8935 ± 0.0189	0.4460 ± 0.0787	0.4643 ± 0.0776	0.8074 ± 0.0317
	Bagging	0.6697 ± 0.0485	0.4928 ± 0.0962	0.8466 ± 0.0417	0.8909 ± 0.0182	0.4039 ± 0.0754	0.4397 ± 0.0718	0.7864 ± 0.0352
Adaptive synthetic sampling	LR	0.7479 ± 0.0523	0.6680 ± 0.0986	0.8278 ± 0.0368	0.9243 ± 0.0209	0.4470 ± 0.0654	0.5331 ± 0.0697	0.8006 ± 0.0346
	KNN	0.7049 ± 0.0552	0.6614 ± 0.1100	0.7484 ± 0.0429	0.9157 ± 0.0250	0.3519 ± 0.0501	0.4576 ± 0.0628	0.7336 ± 0.0371
	SVM	**0.7630** ± **0.0499**	0.6919 ± 0.1008	0.8342 ± 0.0356	0.9301 ± 0.0210	0.4650 ± 0.0578	**0.5531** ± **0.0623**	0.8100 ± 0.0310
	XGBoost	0.6744 ± 0.0554	0.5147 ± 0.1107	0.8340 ± 0.0402	0.8938 ± 0.0215	0.3926 ± 0.0721	0.4417 ± 0.0771	0.7797(0.0352
	Bagging	0.6716 ± 0.0589	0.5033 ± 0.1111	0.8399 ± 0.0429	0.8921 ± 0.0222	0.3983 ± 0.0865	0.4406 ± 0.0858	0.7826 ± 0.0398
Synthetic minority oversampling technique and edited nearest neighbor	LR	0.7245 ± 0.0520	0.7709 ± 0.0937	0.6781 ± 0.0594	0.9356 ± 0.0247	0.3332 ± 0.0470	0.4635 ± 0.0557	0.6939 ± 0.0497
	KNN	0.6813 ± 0.0513	0.7185 ± 0.1060	0.6441 ± 0.0508	0.9188 ± 0.0280	0.2939 ± 0.0355	0.4159 ± 0.0484	0.6568 ± 0.0405
	SVM	0.7306 ± 0.0495	0.7566 ± 0.0956	0.7045 ± 0.0472	0.9344 ± 0.0237	0.3466 ± 0.0431	0.4739 ± 0.0531	0.7134 ± 0.0397
	XGBoost	0.7234 ± 0.0525	0.7061 ± 0.1014	0.7406 ± 0.0453	0.9252 ± 0.0239	0.3608 ± 0.0468	0.4757 ± 0.0575	0.7347 ± 0.0390
	Bagging	0.7020 ± 0.0564	0.6528 ± 0.1067	0.7511 ± 0.0488	0.9138 ± 0.0241	0.3533 ± 0.0593	0.4562 ± 0.068	0.7344 ± 0.0427
Synthetic minority oversampling technique with Tomek inks technique	LR	0.7400 ± 0.0469	0.6419 ± 0.0934	0.8382 ± 0.0343	0.9198 ± 0.0190	0.4522 ± 0.0588	0.5280 ± 0.0623	0.8048 ± 0.0301
	KNN	0.7016 ± 0.0535	0.6366 ± 0.0988	0.7666 ± 0.0504	0.9117 ± 0.0221	0.3639 ± 0.0631	0.4604 ± 0.0674	0.7445 ± 0.0437
	SVM	0.7496 ± 0.0477	0.6557 ± 0.0991	0.8435 ± 0.0394	0.9233 ± 0.0197	**0.4684** ± **0.0658**	0.5422 ± 0.0625	**0.8115** ± **0.0321**
	XGBoost	0.6825 ± 0.0556	0.5019 ± 0.1046	0.8630 ± 0.0351	0.8944 ± 0.0202	0.4341 ± 0.0883	0.4620 ± 0.0861	0.8016 ± 0.0346
	Bagging	0.6696 ± 0.0583	0.4895 ± 0.1227	0.8497 ± 0.0372	0.8910 ± 0.0226	0.4027 ± 0.0753	0.4372 ± 0.0839	0.7884 ± 0.0314

Results expressed as mean ± standard deviation

The applied Classification Algorithms encompassed logistic regression, k-nearest neighbors, support vector machine, extreme gradient boosting and bagging classifier

ROC: receiver operating characteristic; LR: logistic regression; KNN: K-nearest neighbors; SVM: support vector machine; XGBoost: Extreme Gradient Boosting

**Figure 3:**
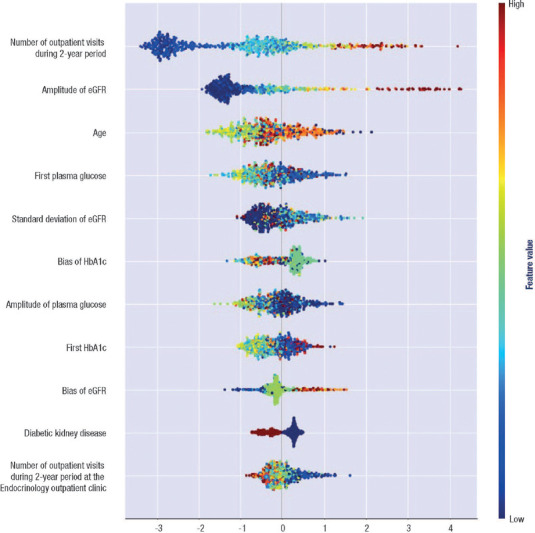
SHapley Additive exPlanations values (features impacts on model outputs). SHapley Additive exPlanations values were computed across the entire dataset using the XGBoost-IHT combination model. Each point on the graph corresponds to a single data observation. The distribution of SHapley Additive exPlanations values is displayed along the horizontal axis through violin plots. Positive SHapley Additive exPlanations values indicate features that contribute to the accurate classification of hospitalization cases, with larger values meaning greater impact. Conversely, negative SHapley Additive exPlanations values are associated with features influencing the prediction of non-hospitalization cases. The color spectrum in the graph represents the actual values of data observations, transitioning from blue to red as the value increases.

**Figure 4:**
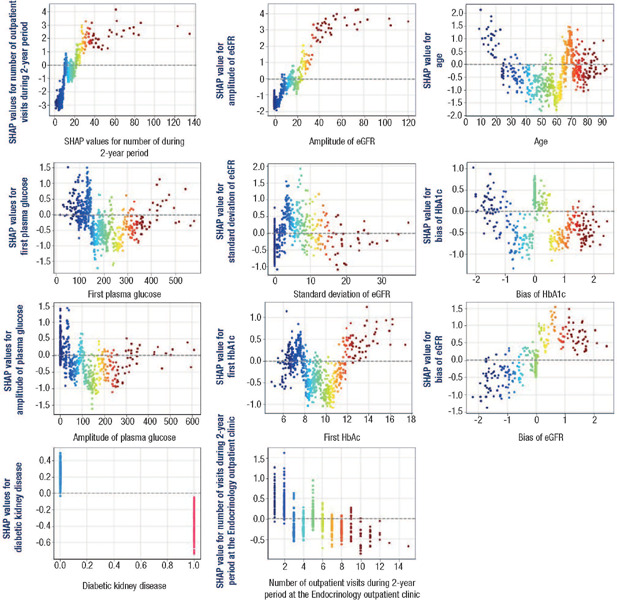
Relationship between the features' real values (displayed along the horizontal axes) and their respective SHapley Additive explanations values (displayed along the vertical axes).

Combining the XGBoost and IHT models yielded the highest sensitivity (value of 0.93) in correctly classifying hospitalization events and an acceptable AUC of 0.72. In addition, it yielded the lowest standard deviation for sensitivity and AUC values, indicating more generalizable results compared to other models. We used SHAP to interpret the features most frequently selected by the XGBoost-IHT combination and provide insights about the importance of each feature (Figure 3) and its effect on the classification result (Figure 4).

eGFR: estimated glomerular filtration rate; HbA1c: glycated hemoglobin.

SHAP: SHapley Additive explanations

Figure 3 allows a visual assessment of the features’ importance for the classification of hospitalization cases. Outpatient visits in the 2-year period and amplitude of eGFR were important hospitalization predictors, as demonstrated by their high importance values in Figure 3. Regarding the outpatient visits in the 2-year period, it is clear that many visits (turning red) are associated with larger SHAP values, i.e., it increases the probability of hospitalization. The amplitude behavior of eGFR feature is similar, suggesting that the greater the difference in exam results, the higher the probability of hospitalization. Although in Figure 3 some features show relatively low importance, it is important to assess their clinical relevance in conjunction with other features and make informed decisions. A case in point is the DKD, which, despite its relatively modest SHAP contribution, is informative due to a shift in SHAP values from negative to positive when transitioning from the presence to absence of DKD.

The graphs of Figure 4 show in a clearer way the relationship between features’ values and the probability of hospitalization. This is because the horizontal axes represent the actual values of each feature, while the vertical axes represent their corresponding SHAP values. Age is the third most important feature in predicting hospitalizations. As shown in Figure 4, SHAP values for age vary according to the patient's age group. For patients under 24 years old, positive SHAP values indicate a higher probability of hospitalization for this age group. In opposition, mostly negative SHAP values associated with the age group between 25 and 65 years old suggest lower probability of hospitalization for this age group. Finally, for patients over 65 years old the relation between feature and prediction is not clear, varying according to other features present in the model.

## DISCUSSION

Considering the limited number of characteristics, we were able to analyze with the available data, the area under the ROC curve reported for the constructed classifier is indeed a considerable achievement for predicting the hospitalization of patients with diabetes, showing that with few easy-to-obtain EMR data it is possible to predict the probability of the patient’s hospitalization, which enables to identify the patient at greatest risk, allowing the allocation of available resources before the outcome occurs.

The results of our study showed that the top three most important features for predicting hospitalization are: number of outpatient visits, amplitude of eGFR, and age (patients under 24 years old and 65 to 70 years old have a higher risk).

The classifier showed that patients who had the highest number of medical consultations during the last 2 years were those with the highest risk of hospitalization. The number of outpatients visits can correlate with the complexity of the patient’s health status, since patients who have more comorbidities or more serious illnesses are those who have a higher number of consultations. A study that evaluated the magnitude and predictors of hospital admission in patients with type 2 diabetes at public hospitals of Eastern Ethiopia showed that medical conditions, including the number of comorbidities and the presence of chronic diabetes complications, are determinant for hospital admission ^(15)^. The number of inpatient healthcare visits can also predict the chance of readmission within 30 days after hospital discharge ^(31)^.

The contribution of eGFR amplitude to predict hospitalization is because patients with varying eGFR may have presented a worsening of their renal function in the last 2 years. A study that evaluated the variability in eGFR in patients with diabetes showed they were at greater risk of major clinical outcomes (major macrovascular events, new or worsening nephropathy, and all-cause mortality) ^(32)^. The study assessed the association between 20-month eGFR variability and the risk of major clinical outcomes in type 2 diabetes among 8,241 patients. Variability in eGFR was calculated from three serum creatinine measurements over 20 months. Compared with low variability, greater 20-month eGFR variability was independently associated with higher risk of the primary outcome with evidence of a positive linear trend (p = 0.015).

As our study showed, age is a known predictor for hospitalization in patients with diabetes. The study revealed that patients who are younger than 24 years old were more likely to be hospitalized, as well as patients between 65 and 70 years old. The fact that younger people are more likely to be hospitalized may represent patients with type 1 diabetes who have diabetic ketoacidosis (DKA). The precipitating factors of DKA were evaluated in a public Brazilian hospital, showing that the mean age of patients with DKA from January 2005 to March 2010 was 26 ± 13 years ^(33)^, remaining at 26.2 ± 14.5 years in the period from April 2010 to January 2017 ^(34)^, treatment noncompliance being the leading precipitating factor in both periods ^(33^,^34)^. A study that evaluated care indicators for patients with diabetes in our country showed that in 2019, worse indicators were observed for younger individuals ^(35)^.

Although patients between 65 and 70 years old had a higher risk of hospitalization than those over 70 years old, we were unable to identify differences in this subgroup. Dennis et al. showed in their predictive model that hospitalized patients with diabetes were older. In addition, age can also predict length of stay ^(36)^ and is an important feature for predicting 1-year mortality ^(37)^.

Our study has limitations. First, from a clinical perspective, our data do not include patients’ medical history such as insulin use, type of diabetes, and duration of diabetes. Second, the database lacks information on important comorbidities and anthropometry. Third, the database had limited sociodemographic information; previous studies showed that low education, low socioeconomic status, high alcohol use, longer diabetes mellitus duration can predict hospitalization ^(15^,^16^,^17)^. Moreover, patients attending our hospital’s outpatient Endocrinology clinic could have been hospitalized in another hospital and this would not have been identified because the search for outcomes was done exclusively in our hospital database.

Despite these limitations, it is essential to consider the financial implications of implementing predictive models in healthcare settings. Implementation costs can vary significantly; according to Al Meslamani ^(38)^, these considerations encompass various expenses, including the initial model development, its real-world implementation, user training, and ongoing maintenance. Cost estimates can vary widely; for instance, simple models may incur operational costs ranging from $60,750 to $94,500, whereas training more complex models can cost tens of millions. The sustainability of funding, coupled with potential secondary savings—such as increased efficiency for providers, reduced hospitalizations, and shorter lengths of stay—is vital for justifying investments in these models. For example, Brisimi et al. revealed that in 2012 the average hospitalization cost in the United States was $9,500. A predictive model with an 81% detection rate could reduce preventive measure costs to just $320 per patient, potentially saving up to $1 billion in avoidable hospitalizations across the Unites States ^(39)^.

## CONCLUSION

The proposed model demonstrated predictive capability and may help identify patients with diabetes who are at higher risk of hospitalization. That allows paying special attention to these patients during outpatient follow-up and identify patients with greatest risk upon arrival at the emergency room, optimizing resources allocation. The factors that most contribute to the prediction are the number of outpatient visits, amplitude of estimated glomerular filtration rate and age (patients under 24 years old and 65 to 70 years old present higher probability).

## Appendix

**Table S1:** Overview of resampling techniques

Techniques	Description	References
IHT	Undersampling technique that removes observations from the majority class that are considered difficult to classify correctly, based on a measure of “instance hardness”. The objective is to keep only the instances that are easiest to classify, so that the model can focus on the most important instances and improve performance in the classification	Smith et al. ^(40)^
RUS	Undersampling technique that reduces the number of observations in the majority class at random until the sample size matches that of the minority class. A major disadvantage is that important information from the sample may be lost due to the random removal of observations	Hanafy et al. ^(41)^
SMOTE	Oversampling technique that involves selecting a random observation from the minority class and then randomly choosing one of its k nearest neighbors. The Euclidean distance between the chosen observation and its nearest neighbor is then multiplied by a random value between zero and 1 and added to the chosen observation, resulting in a synthetic observation. This process is repeated until the desired level of oversampling is achieved. By creating synthetic observations in this way, the model can learn from additional instances of the minority class, leading to better overall performance	Wei et al. ^(42)^ and Chawla et al. ^(43)^
ADASYN	Oversampling technique that generates synthetic instances for the minority class using a density distribution, aiming to create more observations in regions where the density is low. By generating more synthetic instances in these regions, ADASYN adapts the classification decision boundary towards the difficult observations, thereby improving model performance on imbalanced datasets	Wei et al. ^(42)^
SMOTEENN	SMOTEENN is a technique that combines oversampling through the SMOTE technique and undersampling through the ENN technique. First, SMOTE is used to generate synthetic observations for the minority class. Next, ENN is applied to remove noisy observations that may have been introduced during the oversampling process. ENN removes any observation whose class is different from the majority class of its nearest neighbors	Chawlaet al. ^(44)^
SMOTETomek	SMOTETomek combines oversampling through the SMOTE technique and undersampling through the Tomek links technique. First, SMOTE is used to generate synthetic observations for the minority class. Next, Tomek selects random observations from the majority class and identifies any pairs of observations (one from the majority class and one from the minority class) that form a Tomek link, i.e., a pair of points from different classes that are each other’s nearest neighbors. Links are finally removed to help increase the separation between minority and majority classes	Chawla et al. ^(44)^ and Batista et al. ^(45)^

IHT: Instance Hardness Threshold; RUS: Random Under Sampler; SMOTE: Synthetic Minority Oversampling Technique; ADASYN: adaptive synthetic sampling; SMOTEENN: synthetic minority oversampling technique and edited nearest neighbor; ENN: Edited Nearest Neighbors; SMOTETomek: synthetic minority oversampling technique with Tomek links technique.

**Table S2:** Overview of machine learning algorithms for classification

Method	Description	References
LR	LR is a statistical technique used to predict a binary dependent event, and it gets its name from the logistic function it employs, which produces values between zero and one. As a popular probabilistic classification method for binary classification tasks, logistic regression models are obtained by minimizing the regularized negative log-likelihood function to achieve optimal results	Godoy et al. ^(46)^
KNN	KNN works by finding the k nearest observations in the training set to an unclassified observation and assigning the class label of the majority of the k-nearest neighbors to the unclassified observation	Wu et al. ^(47)^
SVM	SVM finds a decision boundary (or hyperplane) between two classes of observations in a dataset by maximizing the margin of separation between the hyperplane and the closest instances, known as support vectors. This leads to a more generalized model that can perform well on new, unseen data. SVM can perform nonlinear classification using the kernel trick, which maps observations onto a higher-dimensional feature space to find the optimal separating hyperplane. Different types of kernel functions can be used with SVM, such RBF, polynomial, and	Wu et al. ^(47)^ and Álvarez-Alvarado et al. ^(48)^
XGBoost	XGBoost is a machine learning algorithm that uses an ensemble of decision trees to make predictions for both regression and classification problems. It stands out from other decision tree algorithms by training a sequence of decision trees that correct the errors of the previous tree. XGBoost optimizes the loss function to minimize prediction errors and improve accuracy, while also incorporating different regularization techniques to prevent overfitting	Grollmuss et al. ^(49)^ and Samih et al. ^(50)^
Bagging Classifier	The Bagging Classifier is an ensemble method that trains multiple independent decision trees on bootstrapped subsets of the data, grows each tree to maximum depth to minimize bias, and combines their predictions to form a robust, lower-variance final model that improves overall performance and reduces overfitting	Bbeiman ^(51)^

LR: logistic regression; KNN: K-nearest neighbors; SVM: support vector machine; RBF: radial basis function; XGBoost: Extreme Gradient Boosting.
